# Certain Effects of Tobacco Smoking

**Published:** 1890-02

**Authors:** 


### On Certain Effects of Tobacco Smoking.

D. A. G. Auld, of Glasgow, thinks that tobacco is responsible for a variety of functional derangements which there is no reason to aver can not terminate in organic disease.

He is convinced that the slightest trace of albumen in the urine is pathological, and that it is frequently induced by preventable causes, and one of these is chronic poisoning by nicotine. He thinks he has certainly traced the disorder in a few cases entirely, and in others partially, to the habit in question.

Another derangement consists in localized fibrillary twitchings, something similar to what is observed in progressive muscular atropy, and perfectly distinct from tremor. The twitchings are often excessive, and occur most frequently about the trunk and upper arms.—*Lancet.*

Hygiene and Physical Culture at Gettysburg.—The parents of the late Dr. Charles H. Graff have donated the sum of $25,000 to the Pennsylvania Hospital, Gettysburg, for the purpose of endowing a professorship of hygiene and physical culture in that institution.

